# Prospective 3D Investigation of Bleb Wall after Trabeculectomy Using Anterior-Segment OCT

**DOI:** 10.1155/2017/8261364

**Published:** 2017-08-29

**Authors:** Fumika Watanabe-Kitamura, Toshihiro Inoue, Sachi Kojima, Kei-Ichi Nakashima, Ayako Fukushima, Hidenobu Tanihara

**Affiliations:** Department of Ophthalmology, Faculty of Life Sciences, Kumamoto University, Kumamoto, Japan

## Abstract

**Purpose:**

We used three-dimensional anterior-segment optical coherence tomography (3D AS-OCT) to evaluate time-dependent posttrabeculectomy changes in bleb wall volume and intensity.

**Methods:**

This prospective observational study included patients with open-angle glaucoma who underwent fornix-based trabeculectomy between January 2012 and October 2012. Twenty-nine eyes met inclusion criteria, and the bleb walls of 22 were amenable to three-dimensional analysis by 3D AS-OCT for 1 year after surgery. The high-intensity volume ratio was calculated as the proportion of the high-intensity region in the total bleb wall. Changes in the high-intensity volume ratio were of high intensity, and parameters influencing the ratio were analyzed using 3D AS-OCT.

**Results:**

The mean high-intensity volume ratios (±SDs) were 43.5 ± 21.4, 44.1 ± 14.8, 41.5 ± 22.6, and 43.2 ± 19.7% at 0.5, 3, 6, and 12 months after trabeculectomy, respectively. When the volume ratios obtained 0.5 and 12 months posttrabeculectomy were compared, four and five eyes exhibited decreases and increases of over 20%, respectively. The volume ratios at 12 months correlated with the intraocular pressure (IOP) at that time (*t* = 2.44, *P* = 0.024) and the bleb wall vascularity score at 12 months (*t* = 5.44, *P* < 0.001).

**Conclusions:**

The high-intensity bleb wall at 12 months posttrabeculectomy reflected the IOP and the bleb wall vascularity at that time.

## 1. Introduction

Lowering of intraocular pressure (IOP) in glaucoma patients reduces the risk of progression of visual field damage; surgery may be required in patient refractory to medication. Trabeculectomy is considered to be a standard surgical modality used to manage IOP, and IOP control is closely related to morphological changes in the filtering bleb [1]. Optical coherence tomography (OCT) is noninvasive and affords high resolution and deep penetration; OCT can thus be used to monitor bleb condition [1]. Recently, three-dimensional clinical images afforded by anterior-segment OCT (3D AS-OCT), an advanced imaging technology, have been obtained by several researchers, including us [2–6].

In previous studies, we used 3D AS-OCT to identify aqueous humor filtration openings on the scleral flaps created by trabeculectomy; these openings were identifiable in more than 95% of filtering blebs [4, 5]. Furthermore, we prospectively investigated time-dependent changes in bleb parameters after trabeculectomy and found that the width of the filtration opening at 0.5 months correlated with the IOP 12 months posttrabeculectomy. Thus, the width of the filtration opening in the early postoperative period was suggested to be potentially prognostic of long-term IOP control. However, any mechanism by which a narrow filtration opening causes later IOP elevation remains unclear. One possibility is that a narrow opening directly controls the future IOP and would thus not correlate with features of the bleb wall and surrounding tissues. Another possibility is that the width of the opening could serve as an index of the extent and rapidity of future bleb scarring, including scarring of the bleb wall. To address this issue, we prospectively observed three-dimensional changes in the intensities of bleb walls using 3D AS-OCT.

## 2. Methods

### 2.1. Ethics, Consent, and Permissions

All procedures adhered to the tenets of the Declaration of Helsinki. This prospective observational study was approved by the Institutional Review Board and Ethics Committee of Kumamoto University and was registered with the University Hospital Medical Information Network Clinical Trials Registry of Japan (ID UMIN000006008; date of access and registration, July 21, 2011). Each patient gave written informed consent prior to study commencement.

### 2.2. Patients

The inclusion criteria were described earlier [6]; all had open-angle glaucoma and underwent fornix-based trabeculectomy which was not combined with other surgery, with application of mitomycin C (MMC), at the Kumamoto University Hospital between January 2012 and October 2012, and their eyes yielded analyzable 3D anterior-segment OCT (3D AS-OCT) images of the filtering blebs 0.5 months after surgery. Patients who had undergone any prior ocular surgery were excluded, with the exception of those who had been treated with phacoemulsification prior to trabeculectomy. When both eyes of a patient met the inclusion criteria, only the eye that was treated first was included in the analysis. When additional glaucoma surgeries (including needling) were required, that eye was excluded from the analysis of time-dependent changes in bleb parameters.

### 2.3. Surgical Procedures

Two experienced surgeons (TI and HT) performed all surgeries in an identical manner. All trabeculectomies employed the same procedure, as described previously [6]. Postoperatively, all patients were prescribed similar topical medical regimens: 1% (*w*/*v*) topical atropine sulfate for 1 week and 0.1% (*w*/*v*) topical betamethasone and 1.5% (*w*/*v*) levofloxacin for approximately 3 months. Laser suture lysis was not performed during the 0.5–12-month observational period after surgery. The surgeons who performed the primary trabeculectomies considered the need for postoperative glaucoma eye drops based on IOP values, the extents of visual field disturbance, and the appearance of the blebs upon slit lamp biomicroscopy. However, the surgeons were blinded to bleb parameters as revealed by 3D AS-OCT. When the IOPs could not be controlled postoperatively by the maximum permissible dose of eye drops, additional glaucoma surgeries were performed.

### 2.4. Data Collection

Each baseline IOP was the average of three measurements taken during three consecutive visits prior to trabeculectomy. At 0.5, 3, 6, and 12 months after trabeculectomy, filtering bleb images (8 × 8 mm square) were acquired via 3D AS-OCT (CASIA; Tomey, Nagoya, Japan) as described previously [4–6]. At each visit, the eyes were examined using a slit-lamp and IOP values measured by Goldmann tonometry between 13:00 and 16:00. Bleb vascularity was assessed via color photography of the anterior ocular segment and classified using the Moorfields Bleb Grading System (http://www.readingcentre.org/Projects/bleb.aspx) at each visit.

As a post hoc analysis, we defined surgical success as an IOP of <21 mmHg (condition A) or <18 mmHg (condition B) with (qualified success) or without (complete success) the use of topical ocular hypotensive medication. Complete failure was defined as hypotony of <4 mmHg. Kaplan-Meier survival curve analyses were used to calculate the ratio of surgical success.

**Figure 1:**
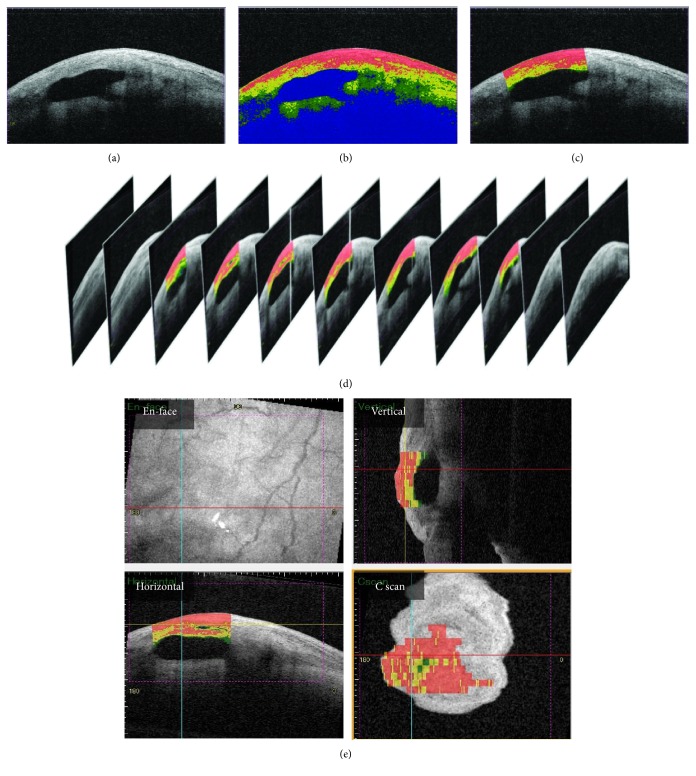
Assessment of bleb wall volume and intensity using 3D AS-OCT. (a) A selected imaging slice from all images of the horizontal raster. (b) A selected imaging slice that was automatically color-coded into four categories based on the optical density: (1) high intensity (red; optical density, 150–255); (2) medium intensity (yellow; optical density, 100–149); (3) low intensity (green; optical density, 50–99); and (4) fluid cavity (blue; optical density, 0–49), using built-in software. (c) A selected imaging slice with color code only in the bleb wall, which was defined to be subjected to analysis by manually drawing lines perpendicular to the scleral flap at the edges of the fluid cavity. (d) Schematic image of the three-dimensional reconstruction of the selected imaging slices after color-coding and bleb wall definition. (e) En-face, vertical, horizontal, and C-scan images after reconstruction with color code in the bleb wall. The red and blue lines indicate the horizontal and vertical axes, respectively. The yellow line represents the *z*-axis of the C-scan images. The volumes of each color-coded category were automatically calculated by reconstructive accumulation of the two-dimensional images, using the built-in software.

Three-dimensional AS-OCT images of filtering blebs were evaluated using CASIA Bleb Assessment Software, version 4.0L (Tomey), as described previously [5, 6]. Briefly, we rotated the 3D AS-OCT images to superimpose the C-scan image planes on the scleral planes. Next, the bleb wall was subjected to three-dimensional analysis. First, 27 imaging slices were automatically selected from all images of the horizontal raster (256 scans; one of every eight slices was thus chosen) using built-in software. Next, the selected images were automatically color-coded into four categories based on optical densities: (1) high intensity (optical density, 150–250); (2) medium intensity (optical density, 100–149); (3) low intensity (optical density, 50–99); and (4) fluid cavity (optical density, 0–49), again using built-in software (Figures 1(a) and 1(b)). Finally, we defined the bleb wall region to be subjected to analysis by manually drawing lines perpendicular to the scleral flap at the edges of the fluid-filled cavities of every selected image (Figure 1(c)). Next, the volume of each color-coded category was automatically calculated via reconstructive accumulation of each two-dimensional image (Figures 1(d) and 1(e)), using the built-in software. The high-intensity volume ratio was calculated as the proportion of high-intensity region in the total bleb wall (with high, middle, and low intensities).

Three independent reviewers (SK, KN, and AF) blinded to clinical backgrounds evaluated all 3D AS-OCT images of the internal structures of filtering blebs and associated findings. The mean values of bleb parameters measured by the three reviewers were subjected to further analysis. Surgeons were blinded for AS-OCT data during the follow-up period, and authors who analyzed the data of AS-OCT were blinded for clinical history until the end of the analysis of all AS-OCT data.

### 2.5. Statistical Analysis

Data were analyzed with the aid of JMP version 8 (SAS Institute, Cary, NC). Changes over time in IOP value, vascularity score, and the volumes of the color-coded bleb-wall categories were compared using the paired Student *t*-test, the Wilcoxon signed-rank test, and the *χ*^2^ test, respectively. Correlations among clinical factors including bleb parameters, vascularity scores, and IOPs were sought by calculating Spearman's correlation coefficients. A probability (*P*) value of less than 0.05 was considered to indicate statistical significance.

## 3. Results

### 3.1. Subjects

**Figure 2:**
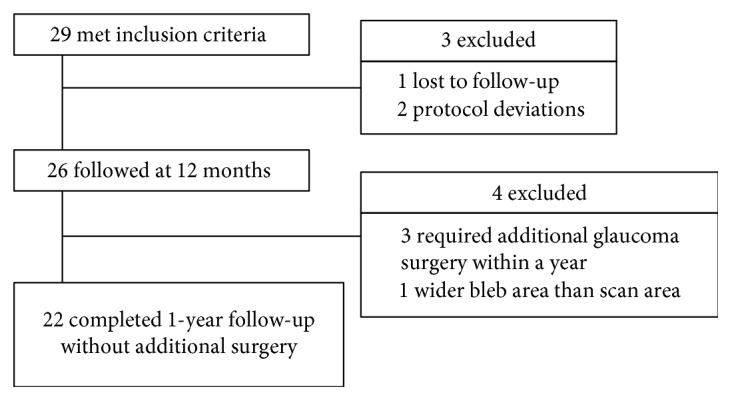
Flowchart showing patient inclusion. One patient of “lost to follow-up” could not come at a given moment, because the patient was admitted to a hospital for a general disease. Two patients of “protocol deviations” presented unanalyzable OCT imaging slices due to involuntary eye movement during the follow-up period.

Twenty-nine eyes (29 patients) met the inclusion criteria; 26 (89.7%) had participated in prior 1-year investigations and have been described previously [6]. Of these, three eyes required additional glaucoma surgeries (needling revision in one eye and retrabeculectomy in two eyes) within 1 year of trabeculectomy, and one eye exhibited a diffuse bleb (the length of the fluid-filled cavity was wider than the scan area of the 3D AS-OCT). Thus, time-dependent changes in bleb wall volume and intensity were analyzed in 22 eyes (75.9%) (Figure 2). Patient characteristics are shown in Table 1. Of those, 9 eyes received laser suture lysis and 10 eyes received bleb massage within 2 weeks after trabeculectomy.

### 3.2. Time-Dependent Changes in IOP Control and Bleb Vascularity after Trabeculectomy

**Figure 3:**
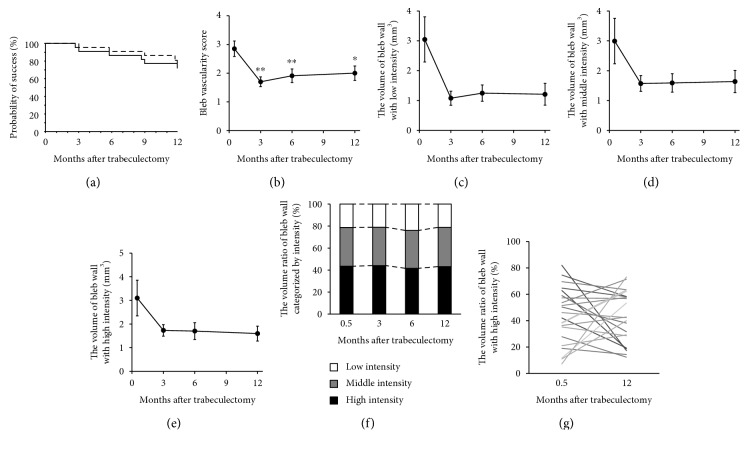
Time-dependent changes in IOP control and bleb parameters. (a) The probability of surgical success (IOP control between 4 and 21 mmHg) based on complete (solid line) and qualified success (dashed line). (b) Time-dependent changes in bleb vascularity score (c, d, e) changes in bleb wall intensity assessed by 3D AS-OCT. Each bleb wall was coded based on optical density: low (c), middle (d), and high (e) intensities. (f) Time-dependent change in the volume ratio of each category was assessed by averaging. (g) Time-dependent change in the high-intensity volume ratio in each eye. ^∗^*P* < 0.05; ^∗∗^*P* < 0.01 compared with the value 0.5 months after trabeculectomy.

Kaplan-Meier survival analyses indicated that the probabilities of success at 1 year were 71.8% and 75.6% based on complete and qualified success under condition A, respectively (Figure 3(a)). The corresponding values under condition B were 71.8% and 71.8%. Posttrabeculectomy, IOP values were 8.3 ± 2.9, 12.4 ± 6.5, 13.8 ± 4.7, and 12.8 ± 5.0 mmHg at 0.5, 3, 6, and 12 months, respectively. A corresponding number of glaucoma eye drops were 0, 0.1 ± 0.6, 0.3 ± 0.9, and 0.6 ± 1.3, respectively. Higher IOP values were observed at 3, 6, and 12 months posttrabeculectomy than at 0.5 months postsurgery (95% CI: 1.64 to 6.54, *P* = 0.002; 95% CI: 2.92 to 6.81, *P* < 0.001; and 95% CI: 2.23 to 6.75, *P* < 0.001, resp.). Although the requirement for glaucoma eye drops (in terms of number) tended to increase over time, no significant difference among the various time points was evident. In contrast, the bleb vascularity scores at 3, 6, and 12 months after surgery were greater than those at 0.5 months (95% CI: −0.59 to −1.85, *P* = 0.002; 95% CI: −0.50 to −1.72, *P* = 0.004; and 95% CI: −0.44 to 0.55, *P* = 0.025, resp.) (Figure 3(b)).

### 3.3. Time-Dependent Changes in Bleb Wall Volumes after Trabeculectomy

**Figure 4:**
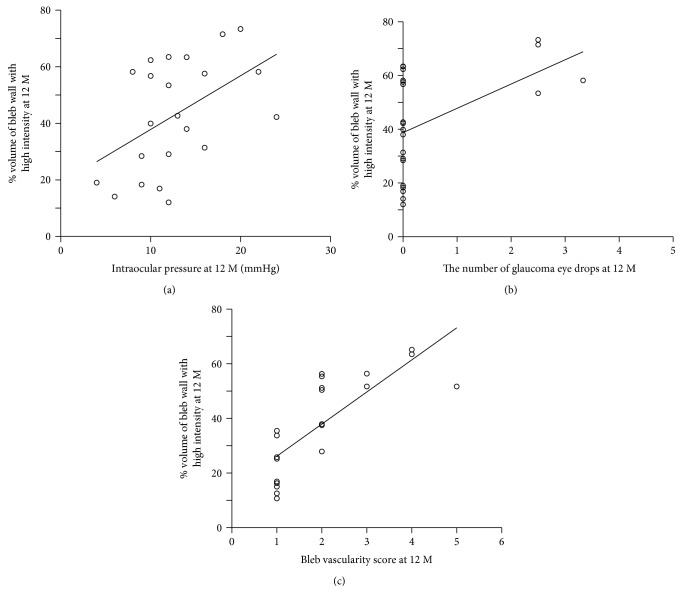
Scatter plots of the 12-month high-intensity volume ratios of bleb walls with 12-month intraocular pressures (IOPs) (a), 12-month requirements for antiglaucoma eye drops (b), and 12-month bleb vascularity scores (c) after trabeculectomy. The lines are linear approximations.

Time-dependent changes in color-coded bleb wall volumes are shown in Figure 4. For all intensity categories, the bleb wall volumes at 0.5 months tended to be greater than those at 3, 6, and 12 months posttrabeculectomy, although the differences were not significant (Figures 3(c), 3(d), and 3(e)). In addition, all average color-coded volume ratios were similar throughout the 12 months posttrabeculectomy (Figure 3(f)). However, time-dependent changes in high-intensity volume ratios were dissimilar (Figure 3(g)). When these ratios were compared 0.5 and 12 months posttrabeculectomy, four and five eyes exhibited decreases and increases (to 12 months) of over 20%, respectively, suggesting that time-dependent changes were both evident and variable.

### 3.4. Correlations among Parameters

At 12 months posttrabeculectomy, the high-intensity volume ratio correlated with the IOP, the number of eye drops needed, and bleb vascularity (*R*^2^ = 0.23, 0.24, and 0.61; *t* = 2.44, 2.55, and 5.44; *P* = 0.024, 0.019, and less than 0.001, resp.) (Figures 4(a), 4(b), and 4(c)).

## 4. Discussion

In the present study, we successfully acquired bleb wall volumes and color-coded these values, reflecting variations in optical density as revealed by 3D AS-OCT. Previously, we performed (only) two-dimensional analyses of time-dependent changes in bleb wall thickness and intensity [6]. To the best of our knowledge, this is the first study to assess bleb walls three dimensionally. The average color-coded bleb wall volume ratios were similar at all time points (Figure 3(e)). However, time-dependent changes in intensity ratios varied among blebs (Figure 3(f)). Also, the ratios obtained 0.5 months posttrabeculectomy did not correlate with those obtained at 12 months. Thus, wound healing and bleb formation differed from eye to eye, indicating that it may be difficult to predict bleb wall status at late postsurgical stages by bleb wall examination soon after trabeculectomy.

In the present study, the high-intensity volume ratio correlated with the IOP, the number of antiglaucoma eye drops required, and bleb vascularity, at the same time points (Figure 3(a)). These data are in good agreement with those of other studies, which found that bleb wall status correlated with IOP control after trabeculectomy [3, 7–13]. The close relationship between IOP and bleb wall status at the same time points is confirmed by the three-dimensional analysis of the present study. We previously showed that oozing of the bleb surface was indicative of a relatively low IOP and low bleb vascularity [13]. Thus, one possible explanation of the relationship between bleb wall status and IOP control may be oozing, which has been suggested to contribute to the lowering of IOP after trabeculectomy. An alternative explanation is that bleb wall status reflects the condition of surrounding tissues, which may in turn be associated with absorbance of aqueous humor into the general circulation.

Bleb wall reflectivity at 2 weeks was suggested to predict bleb functionality 6 months posttrabeculectomy [11]. In the cited study, the subjects were divided into two groups, uniform and multiform, based on qualitative data, such as layer multiplicity, subconjunctival separation, and the presence/absence of microcysts. However, the high-intensity volume ratio at 0.5 months did not correlate with the IOP at 12 months posttrabeculectomy in our present study, indicating that an early quantitative measure of bleb wall intensity was not prognostic of future IOP control. The reason for the between-study discrepancy may be the difference in the manner of assessment; we did not explore multiple layering, subconjunctival separation, or microcyst status in our present work. However, the width of the filtration opening on the scleral flap, measured 0.5 months postsurgery, was prognostic of the IOP 12 months posttrabeculectomy in our previous study [6]. Thus, the data suggest that the width of the filtration opening soon after surgery may predict future changes in bleb wall intensity. That width may serve as an index of the extent and rapidity of future bleb scarring, including scarring of the bleb wall.

All of our patients underwent fornix-based trabeculectomy. As the manner of conjunctival flap creation affects bleb morphology and internal structure, including the positions of filtration openings on the scleral flap [5, 14], it would be interesting to employ 3D AS-OCT to compare time-dependent changes in bleb wall volume and intensity between patients undergoing fornix- and limbal-based trabeculectomy. It is well known that limbal-based trabeculectomy tends to be relatively less vascular and to create a higher bleb than fornix-based trabeculectomy. Additionally, limbal-based trabeculectomy reportedly yields better surgical results in high-risk cases than does the fornix-based approach [15]. Large-scale randomized comparative studies are needed to compare bleb wall status and IOP control between patients undergoing fornix- and limbal-based trabeculectomy.

The limitations of the present study include our relatively small sample size (22 eyes), and a high exclusion ratio (10.3%) before the 1-year observation period was concluded. In addition, the 1-year follow-up results may not be predictive of long-term outcomes; the pathological progression of glaucoma is chronic, extending for decades in many cases. Furthermore, diffuse blebs with fluid-filled cavities wider than the scan length of 3D AS-OCT could not be analyzed. Thus, the data should be interpreted with caution. Larger-scale studies with longer follow-up times are required.

In conclusion, the high-intensity volume ratio 12 months posttrabeculectomy correlated with the IOP and bleb wall vascularity measured at the same time.

## Figures and Tables

**Table 1:** Demographic characteristics patients.

Gender (men/women)	20/2
Mean age ± SD (years old)	64.7 ± 14.1
Mean IOP ± SD (mmHg)	28.1 ± 8.6
Mean number of glaucoma eye drops ± SD	3.1 ± 0.4
Mean MD of Humphrey visual field analyzer ± SD (dB)	−17.53 ± 8.12
Etiology of glaucoma (POAG/EXG)	11/11
Previous cataract surgery (%)	1 (4.6%)

EXG: exfoliation glaucoma; IOP: intraocular pressure; MD: mean deviation; POAG: primary open-angle glaucoma; SD: standard deviation.

## References

[1] Golez E., Latina M. (2012). The use of anterior segment imaging after trabeculectomy. *Seminars in Ophthalmology*.

[2] Miura M., Kawana K., Iwasaki T. (2008). Three-dimensional anterior segment optical coherence tomography of filtering blebs after trabeculectomy. *Journal of Glaucoma*.

[3] Kawana K., Kiuchi T., Yasuno Y., Oshika T. (2009). Evaluation of trabeculectomy blebs using 3-dimensional cornea and anterior segment optical coherence tomography. *Ophthalmology*.

[4] Kojima S., Inoue T., Kawaji T., Tanihara H. (2014). Filtration bleb revision guided by three-dimensional anterior segment optical coherence tomography. *Journal of Glaucoma*.

[5] Inoue T., Matsumura R., Kuroda U., Nakashima K., Kawaji T., Tanihara H. (2012). Precise identification of filtration openings on the scleral flap by three-dimensional anterior segment optical coherence tomography. *Investigative Ophthalmology & Visual Science*.

[6] Kojima S., Inoue T., Nakashima K., Fukushima A., Tanihara H. (2015). Filtering blebs using 3-dimensional anterior-segment optical coherence tomography: a prospective investigation. *JAMA Ophthalmology*.

[7] Sacu S., Rainer G., Findl O., Georgopoulos M., Vass C. (2003). Correlation between the early morphological appearance of filtering blebs and outcome of trabeculectomy with mitomycin C. *Journal of Glaucoma*.

[8] Yamamoto T., Sakuma T., Kitazawa Y. (1995). An ultrasound biomicroscopic study of filtering blebs after mitomycin C trabeculectomy. *Ophthalmology*.

[9] Leung C. K., Yick D. W., Kwong Y. Y. (2007). Analysis of bleb morphology after trabeculectomy with Visante anterior segment optical coherence tomography. *British Journal of Ophthalmology*.

[10] Singh M., Chew P. T., Friedman D. S. (2007). Imaging of trabeculectomy blebs using anterior segment optical coherence tomography. *Ophthalmology*.

[11] Nakano N., Hangai M., Nakanishi H. (2010). Early trabeculectomy bleb walls on anterior-segment optical coherence tomography. *Graefe’s Archive for Clinical and Experimental Ophthalmology*.

[12] Ciancaglini M., Carpineto P., Agnifili L. (2008). Filtering bleb functionality: a clinical, anterior segment optical coherence tomography and in vivo confocal microscopy study. *Journal of Glaucoma*.

[13] Nakashima K., Inoue T., Fukushima A., Hirakawa S., Kojima S., Tanihara H. (2015). Evaluation of filtering blebs exhibiting transconjunctival oozing using anterior segment optical coherence tomography. *Graefe’s Archive for Clinical and Experimental Ophthalmology*.

[14] Hamanaka T., Omata T., Sekimoto S., Sugiyama T., Fujikoshi Y. (2013). Bleb analysis by using anterior segment optical coherence tomography in two different methods of trabeculectomy. *Investigative Ophthalmology & Visual Science*.

[15] Yokota S., Takihara Y., Inatani M. (2015). Limbus- versus fornix-based trabeculectomy for open-angle glaucoma eyes with prior ocular surgery: the Collaborative Bleb-Related Infection Incidence and Treatment Study. *Scientific Reports*.

